# Utilizing the Systemic Immune-Inflammation Index and Blood-Based Biomarkers in Association with Treatment Responsiveness amongst Patients with Treatment-Resistant Bipolar Depression

**DOI:** 10.3390/jpm13081245

**Published:** 2023-08-10

**Authors:** Kyle Decker, Stephen Murata, Nausheen Baig, Sakibur Hasan, Angelos Halaris

**Affiliations:** 1 Department of Psychiatry and Behavioral Neurosciences, Loyola University Chicago Stritch School of Medicine, Loyola University Medical Center, Maywood, IL 60153, USA; kdecker3@luc.edu (K.D.); nbaig5@luc.edu (N.B.); ahalaris@luc.edu (A.H.); 2 Stritch School of Medicine, Loyola University, Maywood, IL 60153, USA; 3 Pine Rest Christian Mental Health Services, Michigan State University, Grand Rapids, MI 49548, USA; 4 Department of Epidemiology and Biostatistics, Michigan State University, East Lansing, MI 48824, USA; hasanmd1@msu.edu

**Keywords:** Systemic Immune-Inflammation Index, inflammation, bipolar depression, treatment resistance, celecoxib, escitalopram, age, neuroprogression, mood disorders, biomarkers

## Abstract

(1) Background: Inflammation is associated with depressive illness and treatment resistance. This study assessed a novel inflammatory index, the Systemic Immune-Inflammation Index (SII), in patients diagnosed with treatment-resistant bipolar depression (TRBDD) before and after treatment with escitalopram (ESC) and celecoxib (CBX) add-on or ESC and placebo (PBO), and compared them to healthy control (HC) subjects. (2) Methods: This is a secondary biological analysis from a double-blind randomized placebo-controlled trial of CBX augmentation in TRBDD. Our subsample with available complete blood count (CBC) data included 52 TRBDD subjects, randomized into an ESC + CBX, (*n* = 29), an ESC + PBO arm (*n* = 23), and an HC group (*n* = 32). SII was calculated from the CBC with differential (SII = platelets x neutrophils/lymphocytes) at baseline and end of treatment (8 weeks). Blood inflammation biomarkers, growth factors, and kynurenine metabolites were determined at both timepoints. Depressive symptom severity was the primary outcome, using the HAMD-17 rating scale score to quantitate treatment response and remission rates. (3) Results: Baseline SII did not discriminate TRBDD from HC, nor was it associated with HAMD-17 score at any timepoint, although it was significantly associated with lower baseline VEGF (*p* = 0.011) and higher week 8 levels of IL1-β (*p* = 0.03) and CRP (*p* = 0.048). Post-treatment HAMD-17 was not independently predicted using baseline SII unless an interaction with age was present (*p* = 0.003 was included), even after relevant adjustments. A similar effect was seen with baseline neutrophils. (4) Conclusions: While SII was not an independent predictor of treatment outcome, elevated baseline SII was a predictor of poor treatment response amongst older patients with TRBDD.

## 1. Introduction

Bipolar disorder (BD), a devastating mental illness affecting over 2.5% of the population, is characterized by mood fluctuations ranging from mania or hypomania to depression with significant subsyndromal symptoms that commonly present between major mood episodes [1]. In the course of bipolar illness, the bipolar depressed phase (BDD) is challenging to manage and, if undertreated, it can lead to worse outcomes, including higher levels of cognitive and functional impairment [2] and increased suicidal risk as compared to patients experiencing hypomania or mania [3,4]. Treatment-resistant bipolar depression (TRBDD) is defined as failed attempts to achieve remission after 8 weeks of at least two separate monotherapeutic treatments or one monotherapy with one combination treatment [5]. The subjective nature in diagnosing BD highlights the unmet need for accurate biomarkers to support the diagnosis, monitor and predict clinical outcomes, and ultimately help arrest the neuroprogressive course of the disease. 

Dysregulation of the immune system has emerged as a major contributor to the pathophysiology of several affective disorders, including BD, with evidence based on elevated levels of circulating immune markers, notably altered release of cytokines, and inflammatory changes in the central nervous system [6,7,8,9]. Chronic inflammation has been correlated to treatment refractoriness in BD, BDD, MDD, and schizophrenia through several biological mechanisms related to cytokine and proinflammatory mediators, alterations in neurotrophins, microglial function, and increased oxidative stress [8,9,10,11,12,13]. A prospective theory linking inflammation and treatment resistance stems from the reconceptualized nature of BD as a neuroprogressive disorder [8,9,14].

A few clinical trials have targeted the immune system in BD subjects through administering treatment with adjunctive inflammatory modulation via the cyclooxygenase-2 (COX-2) inhibitor, celecoxib (CBX), with promising findings of accelerated treatment response due to the constitutional expression of COX-2 in the brain [15,16]. Regarding other psychiatric illnesses, CBX, in addition to mainstay treatment, has shown evidence of improved treatment response and remission in MDD [17] and marked improvement in positive and negative symptoms in schizophrenia [18]. In our main clinical study, we demonstrated the efficacy of CBX add-on therapy in TRBDD where the patient group treated with CBX in addition to the antidepressant, ESC, experienced significantly higher rates of treatment response and remission compared to the group receiving ESC and a placebo add-on in a randomized trial [10]. The present follow-up study was undertaken to further characterize the observed improvement in treatment response in TRBDD patients treated with add-on CBX by means of the Systemic Immune-Inflammation Index (SII) and to seek possible correlations with select inflammatory biomarkers, which may also reflect, at least in part, the presumptive neuroinflammatory process occurring in the CNS. 

The SII is a composite biomarker calculated from the product of absolute neutrophils and platelets, divided by the number of lymphocytes available in the complete blood count (CBC with differential). Developed in 2014 by Hu et al. to predict the prognosis of patients with hepatocellular carcinoma, the SII has been shown to have prognostic value for several solid malignancies [19,20] and cardiovascular disease [21,22], while its use in psychiatric disorders has remained largely under-explored. Precursor indices to the SII, such as the Neutrophil-to-Lymphocyte Ratio (NLR) and Platelet-to-Lymphocyte Ratio (PLR), have been studied in BD, wherein the NLR was significantly elevated in BD versus healthy controls [11,23]. Studies in MDD and BDD have linked higher SII levels to severity of depression and/or mania [23,24,25]. Demir et al. and Kinoshita et al. similarly found a direct relationship between depression severity and the NLR [26,27]. Zhou et al. reported that patients with MDD have a higher SII level compared with healthy controls [24]. Dionisie et al. found the SII to be significantly higher in BDD patients compared to unipolar depression (and healthy controls), as well as higher SII and NLR in the manic phase of BDD [23,28]. The SII is a peripheral inflammation index by extrapolation and may reflect biological processes relevant to the neuroprogression of bipolar depression, such as oxidative stress by way of the neutrophil component, for example [12].

Despite the aforementioned relationships between BDD and peripheral inflammation, to our knowledge, no studies have explored SII in the context of TRBDD. In the associated clinical study, we demonstrated improved response and remission rates in TRBDD patients receiving ESC + CBX compared to ESC + PBO [10]. In this secondary biomarker study, our aim was to characterize the relationship of the SII with the clinical response to treatment in the clinical study. We hypothesized that (1) elevated SII at baseline will discriminate TRBDD from HC subjects; (2) baseline SII is associated with abnormal levels of circulating immune-metabolic biomarkers; and (3), baseline SII is associated with post-treatment clinical outcomes by treatment arm.

## 2. Materials and Methods

### 2.1. Study Population

Males and females aged between 21 and 65 with a diagnosis of BD I or II based on the Diagnostic and Statistical Manual of Mental Disorders IV (DSM-IV) and who met criteria for TRBDD with a minimum score of 18 on the 17-item Hamilton Depression Scale (HAMD-17) were considered for the study. The sample (*N* = 69) included 65.2% female, 65.2% white, mean age 42 years (SD = 12.7). Study participants did not have any other medical diagnosis. A comorbid psychiatric diagnosis, with the exception of anxiety disorder, was an exclusionary criterion. To be classified as treatment resistant, participants had to have previously failed two adequate trials of antidepressants and/or a mood stabilizer or atypical antipsychotic medication, as outlined by the Maudsley Staging Method (MSM) [29]. This is in accord with the definition of TRBD-De, as described in the review article by Fornaro et al. [30]. Patients had to be clinically stable on either a mood stabilizer and/or antipsychotic medication for at least two weeks before entering the study. 

A history of substance use or dependence within 12 months preceding the screening visit was exclusionary. Patients were excluded in the presence of any abnormal routine laboratory examinations, a pain condition including fibromyalgia, history of peptic ulcer, uncontrolled hypertension, anemia, liver disease, kidney disease, arthritis, recurrent migraines, epilepsy, stroke, gum disease, autoimmune disease, pregnant or lactating females, and females taking oral contraceptives. Concurrent use of stimulants, anticoagulant agents, nicotine-containing substances, corticosteroids, or lithium was exclusionary. Celecoxib has the potential to increase lithium blood levels, leading to toxicity [31]. Routine blood analyses were conducted to ensure normal ranges in the CBC, complete metabolic panel, lipid profile, and thyroid function. Urinalysis and urine drug screening were conducted to further exclude participants with underlying infection and/or drug use. Known allergies or hypersensitivities to the study medications and concomitant pharmacologic contraindications were additional exclusion criteria. During the initial screening visit, the study protocol was detailed to potential participants, and written informed consent was obtained as approved by the Institutional Review Board (IRB) of Loyola University Medical Center (LUMC). 

### 2.2. Healthy Controls

The original RCT design for evaluating primary clinical outcomes did not include a healthy control (HC) group precisely matched to the patient group for key demographic parameters [10]. For supplemental molecular analyses, an HC group was utilized from our database (included in the Supplemental Information) to compare against TRBDD subjects. Recruitment for HC subjects was conducted via flyers on the Loyola University Medical Center campus. Volunteers were required to provide written informed consent approved by the IRB before the screening process. Screening and exclusions criteria were similar to the TRBDD groups, with the key difference being a negative history of or concurrent mental illness. Subjects were excluded if they had any current medical conditions or significant history thereof. Regarding mental illness, HCs were excluded if there was any personal or family history in first-degree relatives for substance use and/or mental illness. HAMD-17 scores were required to be less than 5 on the rating scale. Blood samples were obtained once at the initial screening, as HCs did not receive any intervention. Based on our experience, measured values are stable, barring any intercurrent illness. HC subjects were enrolled if their routine laboratory tests fell within the normal range. 

### 2.3. Study Design of Clinical Study

Full details of the study design and study flow chart can be found in our primary study [10]. For ease of reference, the necessary details will be provided here. This was a 10-week, randomized, double-blind, placebo-controlled, two-arm study of TRBDD patients using escitalopram (ESC) in combination with an anti-inflammatory medication, Celecoxib (CBX). It included an initial screening visit, a 2-week minimum washout phase, a 1-week placebo run-in phase, and an 8-week flexible dosing phase. Males and females aged between 21 and 65 who were diagnosed with TRBDD while being mentally and physically capable of consenting to the study were considered. The study was powered for 70 patients (35 in each treatment arm) to complete 8 weeks of active medication with an anticipated 10% dropout rate based on experience with our patient population in the preceding five years. One treatment arm consisted of ESC in combination with CBX (*n* = 26) while the other arm received ESC with PBO (*n* = 21).

Screening visit 1 consisted of a physical exam, blood draws, and urinalysis to obtain CBC, CMP, thyroid function, lipid profile, hCG pregnancy test, and toxicology screen. Subjects were diagnosed with TRBDD through structured interviews using the Mini International Neuropsychiatric Interview (MINI) and the Maudsley Staging Scales. Depression severity and associated symptoms were quantified using Hamilton Rating Scales for Depression (HAMD) and Anxiety (HAMA), Clinical Global Impressions (CGI), and Columbia Suicide Severity Rating Scale (CSSRS). Psychiatric and family histories were obtained through interviewing and with focused questionnaires. After the 1-week placebo run-in phase, subjects were evaluated to effectively rule out placebo responders. Successful placebo non-responders were randomized in a 1:1 fixed assignment ration to receive either ESC + CBX or ESC + PBO. Our approach to stratification included two age groups (21–45 and 46–65) plus binary genders, and group assignment was based on a pharmacy-generated randomization code. The randomization code was generated by the study biostatistician and kept by the institutional pharmacist. Study medications were prepared by the pharmacist, sealed in envelopes, administered to subjects, and returned to the study coordinator after consumption to ensure compliance. 

CBX was dosed at 200 mg twice daily, while ESC was started at 10 mg per day and later titrated up to 10 mg twice per day. However, several exceptions became necessary to optimize clinical response and minimize adverse side effects: in the ESC + CBX arm, 6 patients were dosed at 10 mg of ESC and 1 patient at 30 mg ESC; in the ESC + PBO arm, 3 patients were dosed at 10 mg ESC, and 2 patients were dosed at 30 mg and 40 mg ESC. Along with the study medication, patients were prescribed one or more of the following medications for mood stabilization as indicated: Quetiapine, Lamotrigine, Divalproex sodium, Buspirone, Topiramate, Ziprasidone, Oxcarbazepine, Gabapentin, Carbamazepine, Asenapine, Risperidone, Olanzapine, Aripiprazole, Zolpidem, and Lurasidone. 

A minimum score of 18 on the HAMD-17 scale was required for enrollment. “Responders” to treatment were defined as those whose baseline HAMD-17 scored dropped by at least 50% by week 8 but was still above a score of 7. “Non-responders” were defined as subjects whose HAMD-17 scores dropped less than 50% by week 8. Remission was defined as a score of ≤7 on HAMD-17 at the treatment endpoint (week 8). For participants who dropped out of the study after week 6, their last observation was carried forward in the analysis. 

### 2.4. Laboratory Measurements and Calculation of SII

Subjects returned for follow-up visits at weeks 1, 2, 4, and 8 for blood draws and medication management to assess safety and efficacy. Blood draws occurred consistently between 9 and 10 am to control for diurnal variations. For purposes of this secondary analysis, we utilized the complete blood count with differential (CBC w/diff) at two timepoints, baseline and week 8. The SII was calculated in the following manner:

SII = (absolute platelet count × absolute neutrophil count)/absolute lymphocyte count.

### 2.5. Additional Blood Biomarkers

The following blood biomarkers were analyzed from two timepoints, baseline and week 8, to perform a correlational analysis with SII. Inflammation biomarkers included high-sensitivity C-reactive protein (CRP); the interleukins IL-1A, IL-2, IL-4, IL-6, IL-8, IL-10, IL-18; interferon gamma (IFN-γ); tumor necrosis factor alpha (TNF-α); and the chemokine monocyte chemoattractant protein 1 (MCP-1). The growth factors included epidermal growth factor (EGF) and vascular endothelial growth factor (VEGF). The kynurenine pathway (KP) metabolites included tryptophan (TRP), kynurenine (KYN), Kynurenic acid (KYNA), 3-hydroxykynurenine (3HK), anthranilic acid (AA), xanthurenic acid (XA), picolinic acid (PIC), quinolinic acid (QUIN), and quinaldehyde (QUINA). Biologically pertinent KP metabolite ratios were also calculated, including KYN/TRP, KYNA/KYN, AA/KYNA, 3HK/KYNA, QUIN/PIC, and QUIN/KYNA. 

Plasma samples were analyzed using the Zymutest High Sensitivity CRP enzyme-linked immunosorbent assay (ELISA) kit (Hyphen Biomed^®^, Neuville-sur-Oise, France). This is a highly sensitive “one step” sandwich ELISA technique specific for human CRP. Levels of cytokines and growth factors were measured using a Randox Cytokine and Growth Factors High-Sensitivity Array assay (Randox^®^, London, UK). This is a chemiluminescent immunoassay that operates on a sandwich principle similar to that used in ELISA. Procedures were followed according to the protocols for both assays. Kynurenine pathway metabolites were measured via Ultra Performance Liquid Chromatography/Mass Spectrometry (UPLC-MS), using a Waters Acquity UPLC connected to a Xevo TQ MS triple-quadrupole mass spectrometer, equipped with a Z-spray ESI ion source (Waters, Milford, MA, USA). Separation was carried out using a Kinetex XBC18, 2.6 μm, 2.1 × 150 mm column (Phenomenex, Torrance, CA, USA).

### 2.6. Clinical Outcome Variable

Depression severity was quantitated using the total score of the Hamilton Depression Rating Scale 17 Item (HAMD-17) administered at baseline and the treatment endpoint (week 8). The total HAMD-17 score was used as the primary clinical outcome (continuous variable). Secondary outcome variables were constructed (categorical, dichotomous), including treatment “response”, defined as a ≥50% reduction of the HAMD-17 total score between baseline and week 8, and treatment “remission”, defined as a HAMD-17 total score ≤7 at the treatment endpoint (week 8) regardless of baseline HAMD-17. For the purpose of this study, we only used the HAMD-17 total score, none of the other rating instruments.

### 2.7. Statistical Analysis

Statistical analysis was conducted using R-3.6.3. Associations with *p*-values < 0.05 were considered statistically significant, but *p*-values < 0.1 were also explored based on the exploratory nature of this study. BMI and biomarkers were natural-log-transformed to meet the assumption of normal distributions. We first performed a descriptive analysis of demographic, clinical, and biomarker variables comparing clinical subgroups (using t-tests, ANOVA, and chi-square). In order to screen for relevant covariates for later modelling, we then tabulated the univariate relationships of all variables with HAMD-17, SII, and individual cell counts using linear regressions.

Retrospective power analysis was conducted using an approximate correlation power calculation (arctangh transformation). In the retrospective power analysis, we calculated R = 0.377 based on parameters of *N* = 52, significance level of *p* = 0.05, and power = 0.80. The power calculation yielded R = 0.377, where R^2^ = 0.377^2^ = 0.142. Based on these findings, we would expect a biomarker term to explain 14.2% of the variance in the outcome (HAMD-17 week 8) with the above parameters.

In the subsequent modelling steps, dichotomous variables of sex, treatment arm, response, and remission were treated as dummy-coded variables with respective reference levels: male, placebo, non-responder, and non-remitter. In the first model, we contrasted the SII to HAMD-17 between treatment timepoints (baseline and week 8) using a robust linear mixed model with timepoint as the random variable. In the second model, we used multiple linear regression to describe post-treatment depressive severity (HAMD-17 at week 8) according to the SII, adjusting for demographics, treatment arm, and pre-treatment depressive severity (HAMD-17 at baseline). This model was finalized through the inclusion of significant interactions between the SII and neutrophils with each of the demographic variables, and this final model was depicted visually with interaction plots.

## 3. Results

### 3.1. Sample Characteristics and Group Comparisons (Table 1) 

Our sample with available CBC data consisted of 52 TRBDD subjects and 32 HCs. Compared to HCs, the TRBDD sample had significantly fewer females (*p* = 0.016), elevated BMI (*p* < 0.001), and trending older age (*p* = 0.083). There were no significant group differences in baseline CBC-related biomarkers (neutrophils, monocytes, lymphocytes, or SII) when comparing HC to TRBDD subjects.

The TRBDD sample consisted of *N* = 23 in the placebo (ESC + PBO) arm and *N* = 29 in the treatment arm (ESC + CBX). On group comparison of TRBDD by treatment arm, ESC+ CBX was significantly younger compared to the placebo group (*p* = 0.026), but was similar in regard to sex and BMI. The baseline HAMD-17 was similar (*p* = 0.66) between arms, but the ESC + CBX arm had a significantly lower HAMD-17 score by week 8 (*p* = 0.007). This is congruent with significantly more remitters (*p* = 0.003) and trending toward more responders (*p* = 0.063) in the ESC + CBX arm by week 8. There were no significant differences in CBC-related biomarkers between treatment arms at either timepoint. There were no baseline differences in inflammatory markers between arms, but at week 8, CRP was lower (*p* = 0.004) and IL-1β trended lower (*p* = 0.093) in the ESC + CBX arm. Baseline VEGF trended higher (*p* = 0.057) and FGF trended lower (*p* = 0.074) in the ESC + CBX arm compared to the PBO arm at week 8. There were no differences in KP metabolites or ratios according to treatment arm at baseline, but at week 8, the ESC + CBX arm had a significantly higher KYNA/KYN ratio (*p* = 0.05) and lower AA/KYNA ratio (*p* = 0.038) compared to the PBO arm.

### 3.2. Univariate Relationships of Sample Characteristics with Baseline HAMD-17 (Table 2)

On univariate linear regression of patient characteristics by pre-treatment HAMD-17, there were no significant associations with demographic variables. Baseline HAMD-17 trended toward lower baseline levels of monocytes (*p* = 0.058) and higher MCP-1 at baseline (*p* = 0.087). Meanwhile, baseline HAMD-17 was significantly associated with lower IL-2 at baseline (*p* = 0.035), higher TNF-α at week 8 (*p* = 0.043), and lower QUINA at week 8 (*p* = 0.015).

### 3.3. Univariate Relationships of Sample Characteristics by Baseline SII (Table 3)

Baseline SII trended lower in female subjects (*p* = 0.087). Baseline SII was associated with higher platelet counts (*p* < 0.001), higher neutrophil counts (*p* < 0.001) at both timepoints, and lower lymphocyte counts at baseline only (*p* = 0.02). From the standpoint of inflammatory markers, baseline SII trended toward lower baseline IL-4 (*p* = 0.074) and lower baseline IFN-γ (*p* = 0.089); baseline SII was also associated with biomarkers at week 8, including higher IL-1B (*p* = 0.03) and higher CRP (*p* = 0.048). There was a significant positive association between baseline levels of SII and VEGF (*p* = 0.011). Meanwhile, baseline SII trended toward lower 3-HK at baseline (*p* = 0.082) and toward a higher baseline QUIN/KYNA ratio at baseline and week 8 (*p* = 0.06 and *p* = 0.09, respectively).

### 3.4. Modeling HAMD-17 by SII-to-Timepoint Relationship

The purpose of the first model was to assess whether the relationship between depressive severity and SII contrasts between baseline and week 8. To that end, we constructed a robust linear mixed effects model (Table 4) with the total HAMD-17 score (continuous outcome variable) and SII being dependent on treatment timepoint (main dependent variables), adjusting for age, BMI and sex (dummy-coded categorical variable with ‘male’ as reference category), and BMI and treatment arm (dummy-coded dichotomous variable with reference level ‘ESC + PBO’). BMI was log-transformed to meet the assumption of normality. The SII-to-timepoint relationship was assessed using an interaction variable, SII*Timepoint (where Timepoint was a dummy-coded variable with baseline as the reference level), which was not significant *(p* = 0.498).

### 3.5. Modelling HAMD18 (Week 8) by SII (Baseline)

The purpose of the second model (multiple linear regression, Table 5) was to describe the association between the clinical outcome (HAMD-17 at week 8, continuous outcome variable) and the baseline biomarker (SII baseline, dependent variable of interest) in order to test whether the treatment outcome is modulated by the pre-treatment biomarker. This model was adjusted for age, BMI and sex (dummy-coded categorical variable with ‘male’ as reference category), and treatment arm (dummy-coded dichotomous variable with reference level ‘ESC + PBO’). We also included an adjustment for HAMD-17 (baseline), in order to control for variation in baseline depressive severity. There was no independent significant association (*p* = 0.45) between the SII (baseline) and post-treatment depressive severity (HAMD-17, week 8).

### 3.6. Modelling HAMD-17 (Week 8) by SII (baseline), including Interaction Screen

**Figure 1:**
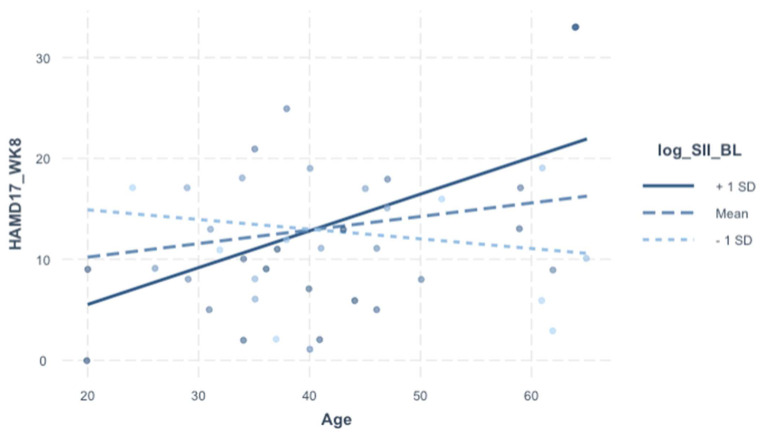
Plot of interaction of HAMD-17 (week 8) according to Age-to-SII (baseline) interaction.

We included an interaction screen with baseline SII (dependent variable of interest) in order to assess whether the effect of the SII (baseline) on HAMD-17 (week 8) is dependent on sex, age, BMI, and treatment arm. There was no direct covariance between the SII (baseline) and demographic variables (see Table 3). Even though there were no independent effects of the SII (baseline) on HAMD-17 (week 8) in the prior model (Table 5), the interaction screen was pursued because sex, age, and BMI are relevant for the SII and depression (both biologically and clinically). The interaction term was not significant for sex or BMI, but it was for age (β = 0.43, 95% CI [0.16–0.70], *p* = 0.003). In the final model (Table 6), the analytically pertinent variable is portrayed visually in Figure 1; even the independent effects of the SII and age were statistically significant; they do not bear explanatory significance in relation to outcome in the context of this interaction model.

### 3.7. Relationships of HAMD-17 with Individual Cell Counts (Table 7 and Table 8)

**Figure 2:**
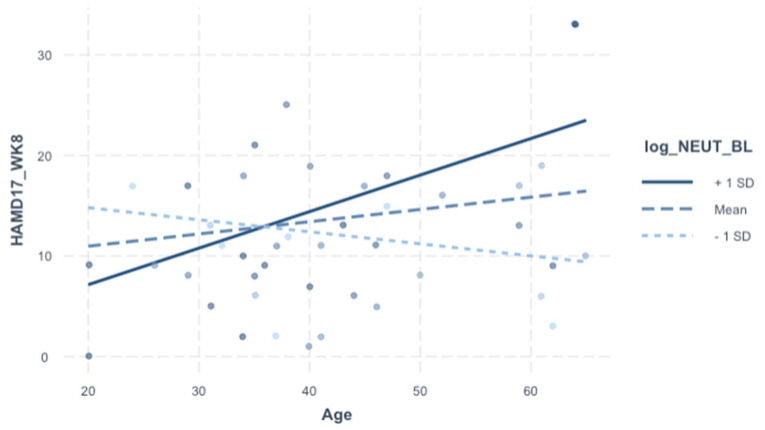
Plot of interaction of HAMD-17 (week 8) according to Age-to-Neutrophil interaction.t.

For exploratory purposes, we included additional analysis into the individual cell counts integral to the SII (neutrophils, platelets, lymphocytes). The rationale was to delineate which cell type may be contributing to the significant SII-to-age interaction on HAMD-17 (Week 8) depicted in Table 6. HAMD-17 (week 8) was significantly associated with age–neutrophils interaction specifically (but not with platelets or lymphocytes), and this relationship survived adjustment for demographics, baseline HAMD-17, and treatment arm (*p* = 0.006) (Table 7 and Table 8, Figure 2).

## 4. Discussion

Growing evidence supports the involvement of inflammation in the pathogenesis of mood disorders resulting from immune dysregulation [7,8,16,23], but few studies have explored the impact of systemic inflammation in BDD, and particularly TRBDD. Based on the notion that adjunctive immune modulation can reverse treatment resistance in TRBDD, which we demonstrated in the primary clinical publication, herein we profiled the novel marker SII in relation to treatment response. We found that pre-treatment SII was associated with treatment response amongst older TRBDD patients, despite our finding that SII did not discriminate TRBDD from HC, and SII was not clearly associated with elevated pro-inflammatory markers.

### 4.1. Associations of SII with TRBDD and Other Blood-Based Biomarkers

We hypothesized that the SII would distinguish TRBDD from HC. In our prior biomarker work in this TRBDD cohort, we found that patients were distinguished from HC by higher blood levels of CRP and IL-1β, suggesting an elevated inflammatory burden in TRBDD compared to the healthy control group [32,33]. However, in the current study, there were no group differences in baseline SII, and there was no independent association between pre-treatment depression severity (HAMD-17 baseline) amongst TRBDD subjects. While these negative findings (on a univariate level) seem discrepant with the expectation of elevated inflammatory burden in TRBDD, it is not entirely clear whether the SII and classical biomarkers of inflammation are directly comparable, from the standpoint of pathophysiological significance. For this reason, one of the reasons for the univariate screen on the SII (baseline) was to establish a reference point with known circulating markers of inflammation or metabolic dysregulation. There were relatively sparse SII-to-cytokine relationships that reached statistical significance (including with CRP and IL-1β), and the few significant associations with the SII were mixed. The SII was associated with lower VEGF (neuroprotective), which is consistent with a chronic pro-inflammatory state, albeit indirectly. However, the SII was significantly associated with lower pro-inflammatory markers, notably IL-4 and IFN-γ, which is paradoxical considering the pro-inflammatory significance of SII in the literature. Meanwhile, SII relationships with KP metabolites were largely non-contributory, although there were notable trends with the neurotoxic KP biomarkers, 3-HK and QUIN/KYNA, albeit in opposite directions. Given the mixed nature of these findings, it is not surprising that SII did not significantly discriminate TRBDD from HC; larger studies are needed to clarify possible significance of SII-to-biomarker relationships and group differences. It is also plausible that the discrepancies may have a biological explanation, such as differences in reflecting chronic vs. acute inflammation.

### 4.2. Pre-Treatment SII Is Associated with Treatment Response, Depending on Age

Despite the finding that the SII (baseline) was not independently associated with categorical (group differences) or continuous (HAMD-17) measures of depression, it became a remarkable predictor of post-treatment outcomes when considered in relation to age. Put simply, in older patients (>40 years), an elevated pre-treatment SII was a poor prognostic marker for post-treatment outcomes (see Figure 1). Why the effect of baseline SII on treatment response is dependent on age remains unclear, especially considering that age was not independently related to baseline SII or HAMD-17 in our sample.

The literature indicates that “chronic non-resolving inflammation” is a function of age [34,35,36]. Chronic non-resolving inflammation is known to be a risk factor for a broad range of age-related conditions, including hypertension, diabetes, atherosclerosis, and cancer [34]. It is understood that a sedentary lifestyle, stress, chronic illness, medical treatment, and aging itself contribute to chronic non-resolving inflammation and potentially treatment resistance [37]. A strong relationship between the SII and age is documented in the literature in other disease processes. A recent study conducted by Hirahara et al. examined the prognostic value of the SII in gastric cancer patients. While the SII was not an independent prognostic factor of overall survival, these investigators found that elderly patients (age > 65) with a high SII (SII > 661.9) had significantly worse overall survival than those with low SII [38]. Similar findings were reported by Hei et al., where an elevated SII was found to be an independent risk factor for advanced endometrial cancer progression in older individuals (age > 55) or postmenopausal patients [39,40]. In the studies described above, the SII indicated utility as a quantitative indicator for poor prognosis and treatment response in older individuals, specifically in the case of certain solid malignancies (gastric cancer and endometrial cancer). Our observations followed a similar trend with regard to age, such that an elevated pre-treatment SII in an older patient portended worse outcomes (i.e., lower HAMD-17 at week 8).

However, since patients with inflammatory illness were excluded from this study, and because age was not independently associated with the SII, it is unlikely that the SII-to-age interaction is explained solely by age-related medical comorbidities. Rather, it is plausible that, because TRBDD is a neuroprogressive illness, age might be a proxy for biological changes associated with the chronic, relapsing–remitting course of bipolar disorder; and therefore, older patients with TRBDD might be more sensitive to the impact of baseline systemic inflammation on clinical prognosis.

### 4.3. Pre-Treatment Neutrophil Count Is Associated with Treatment Response, Depending on Age

We profiled the individual substituents of the SII (baseline neutrophils, platelets, and lymphocytes) in an effort to delineate effects of specific cell types on treatment response, compared to the SII as a whole. We found that, similar to the SII, baseline neutrophil count (but not platelet or lymphocyte counts) predicted treatment response in an age-dependent manner. Although the R^2^ effect size of the SII model (Table 6) was slightly higher than the neutrophil model (Table 8), both were comparable, suggesting that the overall predictive value of SII-to-age on treatment response is largely driven by the neutrophil component. However, the relationships of neutrophils to other biomarkers were notably less significant overall, compared to the SII-to-biomarker relationships.

Because the predictive model of the SII was reducible to one of its cellular components, namely neutrophils, the implication is that propensity for treatment response may be closely related to biological mechanisms surrounding neutrophil activity. Conceptually, the SII is constructed from the ratio of cells involved in the innate immune response divided by the cells involved in the adaptive immune response. Neutrophils are generally associated with the innate immune response, which refers collectively to mechanisms involved in the rapid but non-specific response of a host response to a pathogen (such as chronic low-grade inflammation) [41,42]. Lymphocytes, on the other hand, are generally associated with the adaptive or ‘cell-mediated’ immune system, which refers to more targeted mechanisms tuned to the specific pathogen (such as during a viral infection or even autoimmune illness) [41]. Since baseline neutrophils appear to simulate the predictive value of the SII for treatment response, the general implication is that the innate immune system is an important player in TRBDD prognosis, at least relative to the adaptive immune response. With neutrophils contributing most to the observed effect of SII-to-age on depressive outcomes, it is also worth considering the impact of oxidative stress in the context of innate immunity. The neurotoxic effects of oxidative stress are increasingly implicated in the pathophysiology of mood disorders, and may play a role in treatment-resistant depression within a chronic inflammatory context [8,9,10,12,13]. Thus, when conceptualizing mechanisms involving TRBDD neuroprogression (including age or duration of undertreated illness), future studies might consider dysregulated innate immunity and related pathways.

## 5. Conclusions, Strengths, and Limitations

To our knowledge, this is the first study of the SII in TRBDD. The central finding was an age-dependent predictive association with baseline SII and treatment outcomes. From a biological standpoint, this finding is compatible with the emerging notion that systemic inflammation is important for bipolar neuroprogression. Further, it invites exploration into mechanisms related to innate immunity that may contribute to an underlying vulnerability to treatment refractoriness in TRBDD. From a translational standpoint, the SII seems to bear prognostic value for TRBDD, with the advantages of being affordable and routinely accessible. Further studies are needed to better characterize the precise biological significance of the SII, including its relationships with other known immune-metabolic markers. One peculiarity worth noting is that the predictive value of baseline SII was not significantly dependent on treatment arm, even though the combination of ESC + CBX independently predicted treatment response compared to ESC + PBP. As such, given the design constraints of this secondary post hoc analysis, it is difficult to conclude whether baseline SII (amongst older patients with TRBDD) is a predictive marker for response to CBX augmentation or ESC, which was present in both arms. Larger follow-up studies are needed for further validation of these relationships. Taken together, these findings represent a novel, preliminary step to better clarify TRBDD mechanisms and the impact of systemic inflammation on treatment response.

## Figures and Tables

**Table 1:** Comparison of patient demographic, clinical, and biomarker variables by clinical subgroup.

Patient Characteristics (Whole Cohort)						
Variable	*N*	Overall, *N* = 84	HC, *N* = 32	Treatment Arm (TRBDD Cohort, *N* = 52)		*p*-Value
				ESC + PBO, *N* = 23	ESC + CBX, *N* = 29	
Demographics						
Sex	83					0.016
Male		44 (53%)	11 (34%)	16 (73%)	17 (59%)	
Female		39 (47%)	21 (66%)	6 (27%)	12 (41%)	
Age (years)	74	40 (31, 52)	37 (26, 53)	47 (35, 58)	38 (31, 44)	0.083
BMI (log)	71	3.37 (3.23, 3.49)	3.20 (3.12, 3.34) ^a^	3.44 (3.33, 3.60) ^b^	3.39 (3.29, 3.57) ^b^	<0.001
Clinical characteristics						
HAMD-17 (baseline)	45	24.0 (20.0, 29.0)	-	23.5 (21.0, 26.0)	24.0 (20.0, 30.0)	0.66
HAMD-17 (week 8)	45	10 (7, 17)	-	12 (9, 18)	8 (5, 13)	0.007
Remission	45					0.003
Non-remitter		31 (69%)	-	17 (94%)	14 (52%)	
Remitter		14 (31%)	-	1 (5.6%)	13 (48%)	
Response	45					0.063
Non-responder		17 (38%)	-	10 (56%)	7 (26%)	
Responder		28 (62%)	-	8 (44%)	20 (74%)	
Complete blood count related markers (baseline)						
Platelets	83	5.49 (5.25, 5.60)	5.51 (5.30, 5.61)	5.52 (5.18, 5.62)	5.40 (5.23, 5.57)	0.42
Monocytes	82	−0.92 (−0.92, −0.51)	−0.92 (−0.92, −0.65)	−0.69 (−1.13, −0.40)	−0.69 (−0.92, −0.51)	0.81
Neutrophils	81	1.25 (1.03, 1.57)	1.15 (1.02, 1.53)	1.50 (1.06, 1.68)	1.28 (1.08, 1.49)	0.30
Lymphocytes	82	0.59 (0.47, 0.79)	0.59 (0.52, 0.83)	0.59 (0.47, 0.79)	0.59 (0.52, 0.79)	0.90
Systemic inflammation index (SII)	81	6.17 (5.73, 6.43)	6.19 (5.72, 6.42)	6.26 (5.81, 6.59)	5.96 (5.71, 6.42)	0.55
CBC related markers (week 8)						
Platelets	52	5.40 (5.22, 5.53)	-	5.39 (5.19, 5.54)	5.43 (5.23, 5.53)	0.78
Monocytes	51	−0.69 (−1.20, −0.51)	-	−0.69 (−1.06, −0.43)	−0.69 (−1.20, −0.51)	0.58
Neutrophils	51	1.34 (1.05, 1.54)	-	1.41 (1.05, 1.62)	1.29 (1.08, 1.44)	0.54
Lymphocytes	51	0.64 (0.50, 0.83)	-	0.64 (0.47, 0.79)	0.64 (0.53, 0.84)	0.75
Systemic inflammation index (SII)	48	6.09 (5.61, 6.26)	-	6.14 (5.64, 6.31)	6.03 (5.57, 6.23)	0.36
Inflammatory biomarkers (baseline)						
IL-1α	21	0.69 (0.69, 0.79)	-	0.74 (0.69, 0.80)	0.69 (0.69, 0.69)	0.23
IL-1β	22	0.69 (0.69, 0.88)	-	0.69 (0.69, 0.96)	0.69 (0.69, 0.69)	0.43
IL-2	21	0.69 (0.69, 0.69)	-	0.69 (0.69, 0.69)	0.69 (0.69, 0.69)	0.67
IL-4	26	1.20 (0.81, 1.31)	-	1.21 (0.69, 1.36)	1.20 (1.16, 1.28)	0.94
IL-6	21	1.25 (1.00, 1.44)	-	1.56 (1.01, 1.70)	1.22 (1.00, 1.34)	0.18
IL-8	21	1.40 (1.21, 1.56)	-	1.39 (1.33, 1.64)	1.40 (1.11, 1.56)	0.72
IL-10	21	0.69 (0.69, 1.11)	-	1.10 (0.69, 1.11)	0.69 (0.69, 1.08)	0.18
C-reactive protein	15	1.61 (1.16, 1.95)	-	1.58 (1.22, 1.65)	1.95 (1.19, 2.58)	0.30
IFN-γ	21	0.69 (0.69, 0.69)	-	0.69 (0.69, 0.82)	0.69 (0.69, 0.69)	>0.99
TNF-α	21	1.18 (1.11, 1.40)	-	1.21 (1.12, 1.41)	1.18 (1.08, 1.30)	0.66
Monocyte chemoattractant protein 1 (MCP1)	21	4.59 (4.48, 4.85)	-	4.76 (4.47, 4.90)	4.56 (4.48, 4.71)	0.47
Inflammatory biomarkers (week 8)						
IL-1α	18	0.69 (0.69, 0.69)	-	0.69 (0.69, 0.69)	0.69 (0.69, 0.69)	0.88
IL-1β	30	1.06 (1.00, 1.15)	-	1.12 (1.06, 1.26)	1.02 (1.00, 1.11)	0.093
IL-2	18	0.69 (0.69, 0.69)	-	0.69 (0.69, 0.69)	0.69 (0.69, 0.69)	0.37
IL-4	18	1.16 (0.69, 1.21)	-	1.16 (0.69, 1.28)	1.16 (0.69, 1.21)	0.50
IL-6	20	1.22 (1.07, 1.46)	-	1.19 (1.17, 1.34)	1.26 (1.06, 1.47)	0.77
IL-8	18	1.30 (1.12, 1.71)	-	1.12 (1.06, 2.28)	1.32 (1.13, 1.61)	0.73
IL-10	18	0.87 (0.69, 1.13)	-	0.69 (0.69, 0.69)	1.08 (0.69, 1.14)	0.19
IFN-γ	18	0.69 (0.69, 0.69)	-	0.69 (0.69, 0.69)	0.69 (0.69, 0.69)	0.26
TNF-α	18	1.22 (1.12, 1.44)	-	1.22 (1.16, 2.49)	1.23 (1.11, 1.42)	0.66
C-reactive protein	31	1.47 (0.82, 2.17)	-	2.26 (1.65, 2.66)	1.14 (0.69, 1.79)	0.004
Monocyte chemoattractant protein 1 (MCP1)	18	4.64 (4.08, 4.97)	-	4.21 (4.03, 4.76)	4.82 (4.30, 4.99)	0.59
Growth factors (baseline)						
Fibroblast growth factor (FGF)	15	1.18 (0.88, 1.58)	-	1.36 (1.08, 1.57)	1.11 (0.76, 1.58)	0.73
Epidermal growth factor (EGF)	21	1.28 (0.69, 1.71)	-	1.43 (1.13, 1.61)	1.20 (0.69, 1.71)	0.46
Vascular-endothelial growth factor (VEGF)	31	3.49 (3.36, 3.81)	-	3.41 (3.30, 3.49)	3.63 (3.48, 3.92)	0.057
Growth factors (week 8)						
Fibroblast growth factor (FGF)	9	1.10 (0.69, 1.28)	-	0.69 (0.69, 0.69)	1.28 (0.91, 1.30)	0.074
Epidermal growth factor (EGF)	30	1.53 (1.39, 1.88)	-	1.60 (1.52, 1.91)	1.52 (1.37, 1.84)	0.38
Vascular-endothelial growth factor (VEGF)	30	3.54 (3.37, 3.88)	-	3.47 (3.34, 3.74)	3.56 (3.44, 3.91)	0.67
Kynurenine pathway metabolites (baseline)						
Tryptophan (TRP)	41	9.70 (9.51, 9.79)	-	9.59 (9.45, 9.76)	9.73 (9.58, 9.80)	0.16
Kynurenine (KYN)	41	5.76 (5.56, 5.94)	-	5.74 (5.64, 6.07)	5.76 (5.55, 5.86)	0.71
Kynurenic acid (KYNA)	41	2.24 (2.07, 2.48)	-	2.27 (2.18, 2.44)	2.21 (2.04, 2.48)	0.78
3-Hydroxykynurenine (3HK)	41	2.88 (2.37, 3.28)	-	3.08 (2.43, 3.32)	2.77 (2.35, 3.19)	0.37
Anthran IL-ic acid (AA)	41	1.57 (1.36, 1.79)	-	1.62 (1.43, 2.02)	1.57 (1.31, 1.76)	0.26
Xanthurenic acid (XA)	40	1.54 (1.34, 1.74)	-	1.50 (1.34, 1.77)	1.55 (1.31, 1.72)	0.82
Picolinic acid (PA)	41	3.17 (2.84, 3.33)	-	3.20 (2.69, 3.36)	3.00 (2.84, 3.33)	0.97
Quinolinic acid (QUIN)	41	4.02 (3.76, 4.29)	-	4.04 (3.83, 4.37)	3.98 (3.64, 4.25)	0.39
Quinaldehyde (QUINA)	41	1.28 (1.13, 1.44)	-	1.24 (1.08, 1.38)	1.36 (1.16, 1.44)	0.18
KYN/TRP ratio	41	0.703 (0.701, 0.706)	-	0.705 (0.702, 0.707)	0.702 (0.701, 0.706)	0.25
KYNA/KYN ratio	41	0.704 (0.703, 0.707)	-	0.705 (0.702, 0.706)	0.704 (0.703, 0.708)	0.75
AA/KYNA ratio	41	0.87 (0.81, 0.95)	-	0.92 (0.86, 0.96)	0.84 (0.80, 0.95)	0.14
3HK/KYNA ratio	41	1.40 (1.19, 1.60)	-	1.43 (1.32, 1.64)	1.36 (1.18, 1.54)	0.29
QUIN/PIC ratio	41	1.56 (1.41, 1.76)	-	1.55 (1.47, 1.71)	1.56 (1.39, 1.76)	0.63
QUIN/KYNA ratio	41	2.21 (2.09, 2.49)	-	2.27 (2.14, 2.56)	2.19 (2.02, 2.45)	0.16
Kynurenine pathway metabolites (week 8)						
Tryptophan (TRP)	39	9.63 (9.53, 9.77)	-	9.64 (9.46, 9.74)	9.62 (9.55, 9.83)	0.79
Kynurenine (KYN)	39	5.70 (5.55, 5.90)	-	5.75 (5.58, 6.02)	5.64 (5.55, 5.85)	0.22
Kynurenic acid (KYNA)	39	2.09 (1.97, 2.44)	-	2.03 (1.94, 2.40)	2.20 (1.99, 2.45)	0.33
3-Hydroxykynurenine (3HK)	39	2.69 (2.46, 3.17)	-	2.67 (2.47, 3.26)	2.77 (2.48, 3.00)	0.69
Anthran IL-ic acid (AA)	39	1.63 (1.43, 1.81)	-	1.70 (1.53, 1.98)	1.61 (1.41, 1.74)	0.19
Xanthurenic acid (XA)	35	1.50 (1.39, 1.81)	-	1.44 (1.34, 1.59)	1.54 (1.41, 1.95)	0.11
Picolinic acid (PA)	39	3.03 (2.61, 3.62)	-	2.96 (2.61, 3.59)	3.03 (2.63, 3.62)	0.70
Quinolinic acid (QUIN)	39	3.95 (3.73, 4.22)	-	4.00 (3.65, 4.28)	3.95 (3.77, 4.18)	0.92
Quinaldehyde (QUINA)	39	1.36 (1.16, 1.50)	-	1.28 (1.11, 1.38)	1.41 (1.19, 1.57)	0.13
KYN/TRP ratio	39	0.704 (0.701, 0.706)	-	0.704 (0.702, 0.706)	0.703 (0.701, 0.706)	0.33
KYNA/KYN ratio	39	0.703 (0.702, 0.707)	-	0.702 (0.701, 0.703)	0.705 (0.702, 0.709)	0.05
AA/KYNA ratio	39	0.89 (0.83, 1.00)	-	0.99 (0.88, 1.08)	0.86 (0.82, 0.94)	0.038
3HK/KYNA ratio	39	1.45 (1.21, 1.61)	-	1.46 (1.36, 1.62)	1.30 (1.14, 1.61)	0.24
QUIN/PIC ratio	39	1.50 (1.31, 1.84)	-	1.50 (1.34, 2.30)	1.55 (1.30, 1.76)	0.81
QUIN/KYNA ratio	39	2.23 (2.07, 2.43)	-	2.32 (2.19, 2.63)	2.18 (2.02, 2.31)	0.15

Note: BMI and biomarkers were natural-log-transformed to meet assumption of normalityLegend: TRBDD—treatment-resistant bipolar depression; HC—healthy controls; HAMD-17—Hamilton Depression Rating Scale 17 Item; IL—interleukin. Variable means are accompanied by median (IQR) or frequency (%). Statistical tests utilized werePearson’s Chi-squared test, Kruskal–Wallis rank sum test, Fisher’s exact test. Superscripts were added to indicate significant differences on post-hoc pairwise comparisons where appropriate.

**Table 2:** Univariate relationships of demographic, clinical, and biomarker variables with HAMD-17 (baseline).

Characteristic	*N*	Beta	95% CI ^1^	*p*-Value
Demographics				
Sex (female)	45	−2	−5.8, 1.8	0.3
Age	45	0.05	−0.11, 0.20	0.5
BMI	45	6.7	−2.6, 16	0.2
Clinical characteristics				
Treatment arm (CBX + ESC)	45	−1.2	−5.1, 2.6	0.5
HAMD-17 (week 8)	45	0.16	−0.09, 0.41	0.2
Remission (remitter)	45	−3.5	−7.4, 0.45	0.081
Response (responder)	45	2.9	−0.85, 6.7	0.13
CBC related markers (baseline)				
Platelets	44	3.3	−4.2, 11	0.4
Monocytes	43	−5.3	−11, 0.19	0.058
Neutrophils	43	−2.6	−7.9, 2.7	0.3
Lymphocytes	43	1.5	−5.3, 8.4	0.7
Systemic inflammation index (SII)	43	−1	−4.9, 3.0	0.6
CBC related markers (week 8)				
Platelets	45	4.1	−2.8, 11	0.2
Monocytes	44	−2.9	−7.7, 1.8	0.2
Neutrophils	44	−0.67	−5.5, 4.1	0.8
Lymphocytes	44	1.4	−4.1, 7.0	0.6
Systemic inflammation index (SII)	42	−0.33	−4.4, 3.7	0.9
Inflammatory markers (baseline)				
IL-1α	20	26	−20, 71	0.2
IL-1β	21	2.3	−15, 20	0.8
IL-2	25	−16	−32, −1.3	0.035
IL-4	25	2.5	−6.5, 11	0.6
IL-6	20	2.9	−4.8, 11	0.4
IL-8	30	0.31	−2.2, 2.8	0.8
IL-10	20	−7.8	−20, 4.5	0.2
C-reactive protein	31	1.7	−1.3, 4.7	0.3
IFN-γ	20	−1.7	−11, 7.4	0.7
TNF-α	20	−1.2	−5.0, 2.5	0.5
Monocyte chemoattractant protein 1 (MCP1)	20	6.7	−1.1, 14	0.087
Inflammatory markers (week 8)				
IL-1α	17	0.7	−80, 81	>0.9
IL-1β	17	13	−6.0, 33	0.2
IL-2	17	1.4	−5.3, 8.1	0.7
IL-4	17	−2.4	−14, 9.5	0.7
IL-6	19	3.5	−7.6, 15	0.5
IL-8	17	−1.1	−5.3, 3.1	0.6
IL-10	30	0.57	−28, 29	>0.9
C-reactive protein	12	1.5	−3.0, 6.0	0.5
IFN-γ	17	9.7	−6.6, 26	0.2
TNF-α	30	−1.8	−3.6, −0.06	0.043
Monocyte chemoattractant protein 1 (MCP1)	17	−1.4	−6.9, 4.1	0.6
Growth factors (baseline)				
Fibroblast growth factor (FGF)	14	−0.42	−9.3, 8.5	>0.9
Epidermal growth factor (EGF)	20	0.59	−5.8, 7.0	0.8
Vascular-endothelial growth factor (VEGF)	31	−1.1	−7.3, 5.1	0.7
Growth factors (week 8)				
Fibroblast growth factor (FGF)	8	0.33	−6.0, 6.7	>0.9
Epidermal growth factor (EGF)	17	3.1	−4.0, 10	0.4
Vascular-endothelial growth factor (VEGF)	30	−2.4	−8.3, 3.5	0.4
Kynurenine pathway metabolites (baseline)				
Tryptophan (TRP)	41	−5	−13, 3.2	0.2
Kynurenine (KYN)	41	0.16	−5.8, 6.1	>0.9
Kynurenic acid (KYNA)	41	−1.3	−7.3, 4.8	0.7
3-hydroxykynurenine (3HK)	41	1.2	−2.3, 4.6	0.5
Anthranilic acid (AA)	41	−2.8	−8.3, 2.7	0.3
Xanthurenic acid (XA)	40	−3.8	−10, 2.4	0.2
Picolinic acid (PIC)	41	−1	−4.7, 2.7	0.6
Quinolinic acid (QUIN)	41	0.29	−4.0, 4.6	0.9
Quinaldehyde (QUINA)	41	−4.3	−11, 2.1	0.2
KYN/TRP ratio	41	91	−357, 539	0.7
KYNA/KYN ratio	41	82	−504, 340	0.7
AA/KYNA ratio	41	−3.6	−21, 14	0.7
3HK/KYNA ratio	41	2.9	−3.4, 9.2	0.4
QUIN/PIC ratio	41	0.02	−4.3, 4.3	>0.9
QUIN/KYNA ratio	41	1.5	−4.3, 7.3	0.6
Kynurenine pathway metabolites (week 8)				
Tryptophan (TRP)	39	−4.4	−13, 4.3	0.3
Kynurenine (KYN)	39	−1.7	−8.2, 4.9	0.6
Kynurenic acid (KYNA)	39	−2.4	−8.5, 3.8	0.4
3-hydroxykynurenine (3HK)	39	−1.3	−5.5, 2.9	0.5
Anthranilic acid (AA)	39	−0.68	−5.3, 3.9	0.8
Xanthurenic acid (XA)	35	−2.7	−9.3, 3.9	0.4
Picolinic acid (PIC)	39	−0.2	−3.4, 3.1	>0.9
Quinolinic acid (QUIN)	39	0.78	−4.2, 5.8	0.8
Quinaldehyde (QUINA)	39	−9	−16, −1.9	0.015
KYN/TRP ratio	39	19	−457, 494	>0.9
KYNA/KYN ratio	39	−61	−450, 329	0.8
AA/KYNA ratio	39	0.86	−11, 13	0.9
3HK/KYNA ratio	39	−0.45	−7.0, 6.1	0.9
QUIN/PIC ratio	39	−0.45	−4.6, 3.7	0.8
QUIN/KYNA ratio	39	3.7	−2.7, 10	0.2

Note: BMI and biomarkers were natural-log-transformed to meet assumption of normality. Legend: TRBDD—treatment-resistant bipolar depression; HC—healthy controls; HAMD-17—Hamilton Depression Rating Scale 17 Item; IL—interleukin. ^1^ CI = confidence interval.

**Table 3:** Univariate relationships of SII (baseline) with demographic, clinical, and biomarker variables.

Characteristic	*N*	Beta	95% CI ^1^	*p*-Value
Demographics				
Sex (female)	48	−0.27	−0.57, 0.04	0.087
Age	48	−0.01	−0.02, 0.01	0.3
BMI	47	−0.17	−1.0, 0.66	0.7
Clinical characteristics				
HAMD-17 (baseline)	43	−0.01	−0.03, 0.02	0.6
HAMD-17 (week 8)	43	0	−0.02, 0.02	0.8
Remission (remitter)	43	0.03	−0.31, 0.38	0.8
Response (responder)	43	−0.02	−0.34, 0.30	>0.9
CBC related markers (baseline)				
Platelets	49	0.9	0.40, 1.4	<0.001
Monocytes	49	0.32	−0.09, 0.73	0.12
Neutrophils	49	1	0.79, 1.3	<0.001
Lymphocytes	49	−0.6	−1.1, −0.10	0.02
CBC related markers (week 8)				
Platelets	49	0.51	−0.05, 1.1	0.073
Monocytes	48	0.28	−0.09, 0.65	0.13
Neutrophils	48	0.57	0.27, 0.87	<0.001
Lymphocytes	48	−0.13	−0.61, 0.34	0.6
Systemic inflammation index (SII)	48	0.91	0.73, 1.1	<0.001
Inflammatory markers (baseline)				
IL-1α	21	−1	−4.4, 2.5	0.6
IL-1β	22	−0.56	−1.8, 0.73	0.4
IL-2	21	0.1	−0.41, 0.62	0.7
IL-4	26	−0.6	−1.3, 0.07	0.075
IL-6	21	−0.19	−0.75, 0.38	0.5
IL-8	21	−0.06	−0.43, 0.30	0.7
IL-10	21	0.08	−0.85, 1.0	0.9
C-reactive protein	15	0.14	−0.28, 0.56	0.5
IFN-γ	21	−0.54	−1.2, 0.09	0.089
TNF-α	21	−0.08	−0.36, 0.21	0.6
Monocyte chemoattractant protein 1 (MCP1)	21	−0.39	−1.0, 0.22	0.2
Inflammatory markers (week 8)				
IL-1α	18	1.7	−4.7, 8.1	0.6
IL-1β	30	1.8	0.19, 3.5	0.03
IL-2	30	0.35	−0.89, 1.6	0.6
IL-4	18	0.23	−0.69, 1.2	0.6
IL-6	20	−0.08	−1.0, 0.83	0.9
IL-8	18	0.01	−0.33, 0.35	>0.9
IL-10	18	−0.3	−1.2, 0.63	0.5
C-reactive protein	31	0.24	0.00, 0.48	0.048
IFN-γ	18	0.03	−1.3, 1.4	>0.9
TNF-α	18	−0.07	−0.42, 0.27	0.7
Monocyte chemoattractant protein 1 (MCP1)	18	−0.11	−0.55, 0.34	0.6
Growth factors (baseline)				
Fibroblast growth factor (FGF)	15	0.17	−0.48, 0.83	0.6
Epidermal growth factor (EGF)	21	−0.14	-0.60, 0.31	0.5
Vascular-endothelial growth factor (VEGF)	31	−0.63	−1.1, −0.16	0.011
Growth factors (week 8)				
Fibroblast growth factor (FGF)	9	−0.06	−0.81, 0.69	0.9
Epidermal growth factor (EGF)	30	−0.33	−0.78, 0.13	0.2
Vascular-endothelial growth factor (VEGF)	18	0.04	−0.53, 0.60	0.9
Kynurenine pathway metabolites (baseline)				
Tryptophan (TRP)	39	−0.3	−1.0, 0.37	0.4
Kynurenine (KYN)	39	0.04	−0.45, 0.52	0.9
Kynurenic acid (KYNA)	39	−0.27	−0.75, 0.21	0.3
3-hydroxykynurenine (3HK)	39	−0.24	−0.50, 0.03	0.082
Anthranilic acid (AA)	39	0.14	−0.31, 0.58	0.5
Xanthurenic acid (XA)	38	−0.23	−0.73, 0.27	0.4
Picolinic acid (PIC)	39	0.06	−0.28, 0.40	0.7
Quinolinic acid (QUIN)	39	0.13	−0.22, 0.48	0.5
Quinaldehyde (QUINA)	39	−0.09	−0.62, 0.43	0.7
KYN/TRP ratio	39	5.1	−31, 41	0.8
KYNA/KYN ratio	39	−25	−58, 7.3	0.12
AA/KYNA ratio	39	0.67	−0.71, 2.1	0.3
3HK/KYNA ratio	39	−0.24	−0.76, 0.27	0.3
QUIN/PIC ratio	39	0.05	−0.35, 0.44	0.8
QUIN/KYNA ratio	39	0.43	−0.02, 0.88	0.06
Kynurenine pathway metabolites (week 8)				
Tryptophan (TRP)	37	−0.49	−1.2, 0.18	0.14
Kynurenine (KYN)	37	−0.16	−0.67, 0.36	0.5
Kynurenic acid (KYNA)	37	−0.37	−0.83, 0.10	0.12
3-hydroxykynurenine (3HK)	37	−0.13	−0.46, 0.20	0.4
Anthranilic acid (AA)	37	−0.05	−0.41, 0.30	0.8
Xanthurenic acid (XA)	33	−0.35	−0.84, 0.14	0.2
Picolinic acid (PIC)	37	0.13	−0.14, 0.40	0.3
Quinolinic acid (QUIN)	37	0.01	−0.38, 0.41	>0.9
Quinaldehyde (QUINA)	37	0.01	−0.58, 0.61	>0.9
KYN/TRP ratio	37	6.2	−31, 43	0.7
KYNA/KYN ratio	37	−20	−50, 9.6	0.2
AA/KYNA ratio	37	0.15	−0.80, 1.1	0.8
3HK/KYNA ratio	37	0.08	−0.44, 0.59	0.8
QUIN/PIC ratio	37	−0.12	−0.46, 0.22	0.5
QUIN/KYNA ratio	37	0.42	−0.07, 0.90	0.09

Note: BMI and biomarkers were natural-log-transformed to meet assumption of normality. Legend: TRBDD—treatment-resistant bipolar depression; HC—healthy controls; HAMD-17—Hamilton Depression Rating Scale 17 Item; IL—interleukin. ^1^ CI = confidence interval.

**Table 4:** HAMD-17-to-SII relationships by timepoint (robust linear mixed effects model).

Outcome Variable: HAMD 17 Total			
Predictors	Estimates	CI	*p*
(Intercept)	14.44	−9.97–38.85	0.246
Sex [Female]	−0.73	−3.48–2.01	0.601
Age	0.11	0.00–0.22	0.046
BMI	0.02	−0.22–0.25	0.886
Treatment arm [ESC + CBX]	1.34	−1.42–4.09	0.342
SII	0.44	−3.20–4.08	0.814
Timepoint [Week 8]	−23.63	−55.49–8.22	0.146
SII × Timepoint [Week 8]	1.82	−3.44–7.08	0.498
Random Effects			
σ^2^	33.23		
τ_00Timepoint_	0		
ICC	0		
N_Timepoint_	2		
Observations	84		
Marginal R^2^/Conditional R^2^	0.572/0.572		

Note: BMI and SII (baseline) are log-transformed to meet assumption of normality. Sex, treatment arm, and timepoint are dummy-coded variables with ‘male’, ‘ESC + PBO’, and ‘baseline’ as reference levels, respectively.

**Table 5:** HAMD-17 (week 8) according to SII at baseline (multiple linear regression).

Outcome Variable: HAMD 17 (Week 8)			
Predictors	Estimates	CI	*p*
(Intercept)	−21.11	−75.08–32.87	0.433
Sex [Female]	4.75	0.23–9.27	0.04
Age	0.11	−0.07–0.30	0.216
BMI	3.4	−8.06–14.87	0.551
Treatment Arm [ESC + CBX]	−5.55	−10.24–−0.85	0.022
HAMD-17 (baseline)	0.29	−0.06–0.64	0.1
SII (baseline)	1.75	−2.90–6.40	0.45
Observations	43		
R^2^/R^2^ adjusted	0.332/0.220		

Note: BMI and SII (baseline) are log-transformed to meet assumption of normality. Sex and treatment arm is dummy-coded variables with ‘male’ and ‘ESC + PBO’ as reference levels, respectively.

**Table 6:** HAMD-17 (week 8) according to SII at baseline (multiple linear regression model).

Outcome Variable: HAMD 17 (Week 8)			
Predictors	Estimates	CI	*p*
(Intercept)	87.27	4.18–170.37	0.04
Sex [Female]	4.33	0.30–8.36	0.036
BMI	6.21	−4.15–16.56	0.232
Treatment Arm [ESC + CBX]	−4.66	−8.88–−0.45	0.031
HAMD-17 (baseline)	0.12	−0.20–0.45	0.445
SII (baseline)	−17.12	−29.63–−4.61	0.009
Age	−2.47	−4.10–−0.85	0.004
SII (baseline) × Age	0.43	0.16–0.70	0.003
Observations	43		
R^2^/R^2^ adjusted	0.486/0.384		

Note: BMI and SII (baseline) are log-transformed to meet assumption of normality. Sex and treatment arm is dummy-coded variables with ‘male’ and ‘ESC + PBO’ as reference levels, respectively.

**Table 7:** Univariate relationships of neutrophils (baseline) with demographic, clinical, and biomarker variables.

Characteristic	*N*	Beta	95% CI ^1^	*p*-Value
Demographics				
Sex (female)	48	−0.07	−0.31, 0.16	0.5
Age	48	−0.01	−0.02, 0.00	0.13
BMI	47	−0.12	−0.74, 0.49	0.7
Clinical characteristics				
HAMD-17 (baseline)	43	−0.01	−0.03, 0.01	0.3
HAMD-17 (week 8)	43	0.01	−0.01, 0.02	0.5
Remission (remitter)	43	−0.02	−0.28, 0.23	0.9
Response (responder)		−0.07	−0.30, 0.16	
CBC related markers (baseline)				
Platelets	49	0.22	−0.20, 0.64	0.3
Monocytes	49	0.49	0.21, 0.77	<0.001
Lymphocytes	49	0.12	−0.28, 0.52	0.6
Systemic inflammation index (SII)	49	0.58	0.44, 0.72	<0.001
CBC related markers (week 8)				
Platelets	49	0.18	−0.25, 0.60	0.4
Monocytes	48	0.34	0.07, 0.60	0.014
Neutrophils	48	0.58	0.39, 0.77	<0.001
Lymphocytes	48	0.28	−0.06, 0.63	0.11
Systemic inflammation index (SII)	48	0.59	0.42, 0.76	<0.001
Inflammatory markers (baseline)				
IL-1α	21	−1.1	−4.1, 1.8	0.4
IL-1β	22	−0.39	−1.5, 0.69	0.5
IL-2	21	0.04	−0.41, 0.48	0.9
IL-4	30	−1	−2.1, 0.17	0.091
IL-6	21	0.01	−0.47, 0.49	>0.9
IL-8	21	−0.01	−0.32, 0.30	>0.9
IL-10	21	0.1	−0.69, 0.89	0.8
C-reactive protein	15	0.2	−0.10, 0.51	0.2
TNF-α	21	0.09	−0.14, 0.33	0.4
IFN-γ	21	0.16	−0.41, 0.73	0.6
Monocyte chemoattractant protein 1 (MCP1)	21	−0.36	−0.87, 0.15	0.2
Inflammatory markers (week 8)				
IL-1α	18	−0.32	−6.0, 5.4	>0.9
IL-1β	30	1.1	−0.15, 2.4	0.083
IL-2	18	−0.07	−0.55, 0.40	0.7
IL-4	18	0.35	−0.45, 1.2	0.4
IL-6	20	0.12	−0.68, 0.92	0.8
IL-8	18	−0.04	−0.34, 0.25	0.8
IL-10	30	−1.6	−3.2, −0.07	0.042
C-reactive protein	31	0.03	−0.16, 0.22	0.8
TNF-α	18	−0.05	−0.35, 0.26	0.8
IFN-γ	18	−0.23	−1.4, 1.0	0.7
Monocyte chemoattractant protein 1 (MCP1)	18	−0.05	−0.45, 0.34	0.8
Growth factors (baseline)				
Fibroblast growth factor (FGF)	15	0.01	−0.50, 0.52	>0.9
Epidermal growth factor (EGF)	21	−0.12	−0.50, 0.27	0.5
Vascular-endothelial growth factor (VEGF)	31	−0.1	−0.49, 0.30	0.6
Growth factors (week 8)				
Fibroblast growth factor (FGF)	9	0.2	−0.29, 0.70	0.4
Epidermal growth factor (EGF)	18	−0.07	−0.57, 0.43	0.8
Vascular-endothelial growth factor (VEGF)	30	−0.03	−0.38, 0.32	0.9
Kynurenine pathway metabolites (week 8)				
Tryptophan (TRP)	39	0.08	−0.44, 0.60	0.7
Kynurenine (KYN)	39	0.3	−0.07, 0.66	0.1
Kynurenic acid (KYNA)	39	−0.06	−0.44, 0.32	0.7
3-hydroxykynurenine (3HK)	39	−0.09	−0.31, 0.12	0.4
Anthranilic acid (AA)	39	0.08	−0.26, 0.43	0.6
Xanthurenic acid (XA)	38	−0.06	−0.45, 0.33	0.8
Picolinic acid (PIC)	39	0.18	−0.08, 0.44	0.2
Quinolinic acid (QUIN)	39	0.12	−0.15, 0.38	0.4
Quinaldehyde (QUINA)	39	0.18	−0.22, 0.58	0.4
KYN/TRP ratio	39	10	−18, 38	0.5
KYNA/KYN ratio	39	−22	−48, 3.0	0.082
AA/KYNA ratio	39	0.17	−0.92, 1.3	0.8
3HK/KYNA ratio	39	−0.12	−0.53, 0.28	0.5
QUIN/PIC ratio	39	−0.05	−0.35, 0.26	0.7
QUIN/KYNA ratio	39	0.22	−0.14, 0.58	0.2
Kynurenine pathway metabolites (week 8)				
Tryptophan (TRP)	37	−0.26	−0.78, 0.26	0.3
Kynurenine (KYN)	37	−0.04	−0.43, 0.36	0.9
Kynurenic acid (KYNA)	37	−0.19	−0.56, 0.17	0.3
3-hydroxykynurenine (3HK)	37	−0.06	−0.32, 0.19	0.6
Anthranilic acid (AA)	37	0	−0.27, 0.27	>0.9
Xanthurenic acid (XA)	33	−0.31	−0.66, 0.05	0.091
Picolinic acid (PIC)	37	0.18	−0.02, 0.38	0.069
Quinolinic acid (QUIN)	37	−0.03	−0.33, 0.27	0.8
Quinaldehyde (QUINA)	37	0.15	−0.31, 0.60	0.5
KYN/TRP ratio	37	5.8	−22, 34	0.7
KYNA/KYN ratio	37	−14	−37, 8.7	0.2
AA/KYNA ratio	37	0.24	−0.48, 1.0	0.5
3HK/KYNA ratio	37	0.03	−0.36, 0.43	0.9
QUIN/PIC ratio	37	−0.22	−0.47, 0.03	0.08
QUIN/KYNA ratio	37	0.18	−0.21, 0.56	0.4

Note: ^1^ CI = confidence interval.

**Table 8:** HAMD-17 (week 8) according to neutrophils at baseline (multiple linear regression).

Outcome Variable: HAMD 17 (Week 8)			
Predictors	Estimates	CI	*p*
(Intercept)	7.51	−34.18–49.20	0.717
Sex [Female]	3.46	−0.54–7.47	0.088
Age	−0.67	−1.25–−0.09	0.024
BMI	7.43	−3.18–18.05	0.164
Treatment arm [ESC + CBX]	−4.58	−8.80–−0.35	0.035
HAMD-17 (baseline)	0.13	−0.21–0.47	0.449
log Neutrophils (baseline)	−21.05	−39.08–−3.03	0.023
Age × Neutrophils (baseline)	0.6	0.18–1.02	0.006
Observations	43		
R^2^/R^2^ adjusted	0.476/0.371		

Note: BMI and neutrophils (baseline) are log-transformed to meet assumption of normality. Sex and treatment arm is dummy-coded variables with ‘male’ and ‘ESC + PBO’ as reference levels, respectively.

## Data Availability

Not applicable.
